# Transcriptome profiling reveals the underlying mechanism of grape post-harvest pathogen *Penicillium olsonii* against the metabolites of *Bacillus velezensis*

**DOI:** 10.3389/fmicb.2022.1019800

**Published:** 2023-01-18

**Authors:** Tingfu Zhang, Guoqin Wen, Bo Song, Zhenyong Chen, Shijiao Jiang

**Affiliations:** Key Laboratory of Southwest China Wildlife Resources Conservation, School of Life Sciences, China West Normal University, Nanchong, Sichuan, China

**Keywords:** post-harvest pathogen, *penicillium olsonii*, *bacillus velezensis*, biocontrol, *vitis vinifera*

## Abstract

**Introduction:**

Pathogen infection influences the post-harvest shelf life of grape berries. In a preliminary study, metabolites produced by *Bacillus velezensis* significantly inhibited the growth of the grape postharvest pathogen *Penicillium olsonii*.

**Methods:**

To investigate the mechanism of interaction between *B. velezensis* and *P. olsonii*, a draft genome was generated for *P. olsonii* WHG5 using the Illumina NovaSeq platform, and the transcriptomic changes in WHG5 were analyzed in response to the exposure to *B. velezensis* metabolites (10% v/v).

**Results:**

The expression levels of genes associated with sporulation, including *GCY1*, *brlA*, and *abaA*, were down-regulated compared with those of the control. In addition, spore deformation and abnormal swelling of the conidiophore were observed. The expression of crucial enzymes, including fructose 2,6-bisphosphate and mannitol-2-dehydrogenase, was down-regulated, indicating that the glycolytic pathway of WHG5 was adversely affected by *B. velezensis* metabolites. The KEGG pathway enrichment analysis revealed that glutathione metabolism and the antioxidant enzyme system were involved in the response to *B. velezensis* metabolites. The down-regulation of the pathogenesis-related genes, *PG1* and *POT1*, suggested that *B. velezensis* metabolites decreased the pathogenicity of *P. olsonii*. *B. velezensis* metabolites disrupted the homeostasis of reactive oxygen species in *P. olsonii* by affecting glucose metabolism, resulting in spore deformation and disruption of growth. In addition, the expression of key pathogenesis-related genes was down-regulated, thereby reducing the pathogenicity of *P. olsonii*.

**Disscusion:**

This study provides insights into the responses of *P. olsonii* to *B. velezensis* metabolites and identifies potential target genes that may be useful in biocontrol strategies for the suppression of post-harvest spoilage in grapes.

## Introduction

Table grapes (*Vitis vinifera* L.) are among the most popular fresh fruit worldwide and have a relatively long post-harvest shelf life. However, pathogen infection strongly influences the post-harvest shelf life of grape berries (He et al., 2019). Pathogenic fungi, such as *Botrytis cinerea*, *Penicillium* spp., and *Aspergillus* spp., can severely shorten the storage longevity of table grapes and cause major post-harvest losses (Zhang et al., 2021b). In addition, *Penicillium* spp. can produce mycotoxins, of which many are classified as carcinogenic and can adversely affect human health, especially causing damage to the kidneys and the liver (Schmidtke et al., 2019). Controlling post-harvest infection of grape berries by pathogens is mainly dependent on the post-harvest application of synthetic chemical fungicides, which is considered the most economical and effective approach to suppress post-harvest spoilage. However, the use of synthetic chemical fungicides is increasingly prohibited owing to their toxicological risks to human health and the environment, as well as the increasing prevalence of resistance to antimicrobial agents among pathogenic strains (Romanazzi et al., 2012; Pertot et al., 2017; Zhang et al., 2020). The exploration of alternatives to synthetic chemical applications for post-harvest disease management has become an important research focus in recent years (Wang et al., 2019).

Biological control methods have shown great potential and are recognized as effective strategies for the control of post-harvest diseases, especially using antagonist microorganisms (biological control agents) and their metabolites (Jiang et al., 2018; Yu et al., 2020; Chaves-Gómez et al., 2021). Biological control agents have the ability to inhibit post-harvest deterioration and provide efficient eco-compatible biocontrol of post-harvest diseases, on account of their broad-spectrum antimicrobial activity and reduced risk of emergence of resistant fungal strains. *Bacillus* strains provide versatile biological control against plant pathogens owing to their diverse antagonistic mechanisms, which include competition for space and nutrients, antibiosis through secretion of compounds exhibiting antimicrobial activities, secretion of enzymes that degrade the plant pathogen cell wall, and stimulation of host resistance (Shafi et al., 2017; Fira et al., 2018; Dimkić et al., 2022).

Cyclic lipopeptides produced by *Bacillus* strains are the most prominent bioactive compounds for plant protection (Falardeau et al., 2013; Liu et al., 2014; Mora et al., 2015). Members of the surfactin family can induce resistance in diverse hosts against various diseases by stimulating plant immune responses (Hafeez et al., 2019; Park et al., 2019). Surfactins play an important role in the suppression of bacterial fruit blotch in grapes and show antiviral and antibacterial activities (Li et al., 2019). *Bacillus velezensis* KOF112 significantly up-regulates the expression of genes encoding class IV chitinases and β-1,3-glucanase in grape leaves (Hamaoka et al., 2021). *Bacillus cereus* NRKT stimulates an increase in the resveratrol content in the skin of grape berries, which is an important mechanism for the induction of disease resistance (Aoki et al., 2017). Thus, bioactive molecules produced by *Bacillus* spp. may act as elicitors of plant-induced systemic resistance. However, the defense responses tend to be specific to certain plant species or host–pathogen systems. For example, *Bacillus subtilis* BBG111 induces systemic resistance in rice (*Oryza sativa* L.) against *Rhizoctonia solani* but not in grapevine (Chandler et al., 2015). *Bacillus altitudinis* GLB194 and *Bacillus pumilus* GLB197 have strong inhibitory activities against grape downy mildew, and *Bacillus cereus* NRKT increases the resveratrol content in grape berries and reduces the incidence of grape anthracnose (Aoki et al., 2017). Thus, the antimicrobial activity of a strain may differ according to the plant–pathogen interaction.

Therefore, unraveling the mode of action in each plant pathosystem is necessary, and other mechanisms of *Bacillus* biocontrol, such as the strong and broad-spectrum antifungal and/or antibacterial activity, also deserve attention. For example, *Bacillus amyloliquefaciens* inhibits the growth of 13 fungal pathogens of grapes, including *Alternaria alternata*, *Penicillium verrucosum*, *Bacillus cinerea*, *Fomitiporia mediterranea*, *Fusarium oxysporum*, *Lasiodiplodia theobromae*, and *Phoma glomerata* (Alfonzo et al., 2012). The metabolites produced by *B. subtilis* in liquid culture inhibit spore germination and hyphal growth of certain pathogenic fungi (Jiang et al., 2014). *B. velezensis* secretes fungal cell wall-degrading enzymes, which is an important mechanism for inhibiting pathogens and it deserves further research (Ye et al., 2018). Overall, although *Bacillus* species exhibits well-defined antifungal activity and induces host-defense responses against plant pathogens, the mechanism by which *Bacillus* inhibits pathogen growth *in vitro* requires further study and may contribute to the biological control of plant diseases.

Our previous study provided the first report of the fungal pathogen *Penicillium olsonii* causing postharvest fruit rot of grapes (Zou et al., 2022). In a preliminary study, we observed that the growth of *P. olsonii* was significantly inhibited by metabolites produced by a biocontrol strain of *B. velezensis*. Our aims in the present study were to (1) examine the response of *P. olsonii* to metabolites produced by *B. velezensis*, (2) generate a draft genome for *P. olsonii* WHG5 as a tool for further genetic analysis, and (3) elucidate the mechanisms of *B. velezensis* antifungal activity on *P. olsonii*. The results provide insight into the potential of metabolites produced by *B. velezensis* for effective biocontrol of post-harvest fruit rot to enhance the storage longevity of grape berries.

## Materials and methods

### Microbial stains

*Penicillium olsonii* WHG5 was isolated from rotting berries of grape (*V. vinifera* “Crimson”) using the single spore isolation technique developed by Chomnunti et al. (2014). The identification and pathogenicity of WHG5 were reported previously (Zou et al., 2022). A WHG5 cell suspension was generated by washing the cultures grown for 72 h at 28°C on potato dextrose agar medium (PDA; 200 g fresh potato tuber, 20 g glucose, 15 g agar, 1 L distilled water) with sterile distilled water. *B. velezensis* isolate SB023 was obtained from the China General Microbiological Culture Collection Center, Beijing, China. An SB023 cell suspension was generated by culturing a single colony for 48 h at 32°C with shaking at 200 rpm in nutrient broth (NB; 20 g glucose, 5 g yeast extract, 10 g peptone, 5 g NaCl, 1 L distilled water, pH 6.5–6.7). All cell suspensions were mixed with 50% (v/v) glycerin solution (1:1, v/v) and stored at −80°C.

### Metabolite exposure treatment

A sample (100 μl) of the preserved SB023 cell suspension was activated in 10 ml NB by incubation for 48 h at 32°C with shaking at 200 rpm and then adjusted to approximately 1 × 10^8^ CFU/ml as measured with a hemocytometer. An aliquot (1 ml) of the cell suspension was inoculated into 100 ml NB, incubated under the same conditions, and then the culture was centrifuged at 10,000 *g* for 15 min. The cell-free supernatant was filtered through a Millex-HV filter (0.22 mm, 13 mm diameter; Millipore, Billerica, MA, USA) to obtain the sterile *B. velezensis* liquid-culture metabolites.

The WHG5 cell suspension was activated in 10 ml potato dextrose broth (PDB; PDA without agar) by incubation for 24 h at 28°C with shaking at 200 rpm, and then adjusted to approximately 1 × 10^8^ CFU/ml, as measured with a hemocytometer. An aliquot (1 ml) of the culture was added to 100 ml PDB supplemented with 10% (v/v) *B. velezensis* metabolites. An identical volume of PDB supplemented with the equivalent volume of sterile distilled water served as the control. All liquid cultures were incubated for 5 days at 28°C with shaking at 200 rpm and then were used for DNA and RNA extraction. All experiments were repeated three times with three replicates.

### Whole-genome sequencing analysis

Genomic DNA was extracted using a fungal DNA kit (Omega Bio-Tek Inc., Norcross, GA, USA) in accordance with the manufacturer’s instructions. Strains were sequenced and the reads were assembled (by Nanjing Personal Gene Technology Co., Ltd., Nanjing, China) using the following procedure. The genomic DNA was sheared using a g-TUBE (Covaris, Inc., Woburn, MA, USA). A library of 400-bp paired-end reads was constructed in accordance with the standard Illumina TruSeq DNA sample preparation guide and sequenced using the Illumina NovaSeq platform (Illumina, Inc., San Diego, CA, USA) with a read length of 150 bp. The FastQC tool (Brown et al., 2017) was used for quality control on the high-throughput sequence data. High-quality sequence data were *de novo* assembled with A5-miseq v20150522 (Coil et al., 2015) and SPAdesv3.9.0 (Bankevich et al., 2012). The genome assembly was further proofread using Pilon v1.18 (Walker et al., 2014), and the integrity was assessed using BUSCO (Simão et al., 2015). Ultimately, a whole-genome assembly was obtained without redundancy.

Assembled scaffolds were processed using RepeatMasker v4.0.5 and RepeatModeler v1.0.4 software to predict genes (Tempel, 2012). Thereafter, tRNA and rRNA were predicted using rRNAscan-SE v1.3.1 (Lowe and Eddy, 1997) and RNAmmer v1.2 (Lagesen et al., 2007), respectively. Predictions of other non-coding RNAs were mainly obtained by comparison with the Rfam database (Griffiths-Jones, 2006). To improve the accuracy of gene prediction, Augustus v3.03, glimmerHMM v3.0.1, and SNAP software were used for the *de novo* prediction of genetic models in the WHG5 genome (Korf, 2004; Majoros et al., 2004; Stanke and Morgenstern, 2005). Exonerate v2.2.0 software^1^ was used for the prediction of homology among related species. EVidenceModeler vr2012-06-25 software was used to integrate the *de novo* prediction results and the homology prediction results for related species (Haas et al., 2008).

The hmmscan (v3.1b2) and BLASTP (v2.5.0+) tools were used to predict the presence of CAZy enzyme and cytochrome P450 genes in the genome sequence, respectively (Finn et al., 2011). Predicted proteins were compared with the NCBI nr database (release 2017.10.10) using DIAMOND (v0.9.10.111) software (Buchfink et al., 2015) and then functionally annotated using the GO, eggNOG, KEGG, and Swiss-Prot databases. Completeness of genome annotation was estimated using the BUSCO Fungi datasets. Single-copy ortholog sequences were identified with OrthoFinder v2.0 (Emms and Kelly, 2019) and then aligned with the Operon Database v10.1 (Zaidi and Zhang, 2016) using DIAMOND (v0.9.10.111) software. After the removal of identical sequences, the remaining sequences with an E value of <10^–5^ and a coverage of ≥50% were used to construct a multiple sequence alignment using MAFFT software (Katoh and Toh, 2008). Finally, the aligned sequences were spliced according to species and a dendrogram was constructed using the neighbor-joining method with IQtree (Nguyen et al., 2015).

### Transcriptome analysis

Total RNA was extracted using a fungal RNA kit (Omega Bio-Tek Inc., Norcross, GA, USA) following the manufacturer’s instructions. The concentration and purity of the RNA were determined using a NanoDrop 2000 spectrophotometer (Thermo Fisher Scientific Inc., Waltham, MA, USA) and an Agilent 2100 Bioanalyzer (Agilent Technology Inc., Palo Alto, CA, USA), respectively. The cDNA libraries with an average insert size of 380 bp were prepared using the Illumina mRNA-seq sample preparation kit following the manufacturer’s instructions and evaluated with an Agilent 2100 Bioanalyzer. The cDNA libraries were sequenced and analyzed (by Nanjing Personal Gene Technology Co., Ltd., Nanjing, China) in accordance with the Illumina standard protocol. The RNA-sequencing (RNA-seq) reads were aligned to the WHG5 genome sequence assembled in this research.

All read alignment positions of each paired-end read were mapped against the genome sequence and exon splicing junctions using HISAT2 software with the BWT algorithm (Sirén et al., 2014). Read counts per gene were determined from the primary read alignments using HTSeq and then normalized as fragments per kilobase per million mapped reads (FPKM). The read count files with FPKM > 1 were analyzed with the “DESeq2” R package (Love et al., 2014) to identify differentially expressed genes (DEGs). Absolute log_2_ fold change > 1 and statistical significance (*P*-value < 0.05) were used as the criteria to identify the DEGs.

A gene ontology (GO) enrichment analysis was performed using the topGO Bioconductor package. All DEGs were mapped to corresponding GO terms and the number of genes in each term was calculated. The GO terms with significant enrichment of DEGs compared with the genomic background were defined using the hypergeometric test. The GO terms with a false discovery rate of (calculated *P*-value after correction) ≤ 0.05 were defined as significantly enriched DEG GO terms. This analysis determined the main biological functions of the DEGs (Mi et al., 2016).

A Kyoto Encyclopedia of Genes and Genomes (KEGG) pathway enrichment analysis was performed using the KEGG database.^2^ The selected DEGs were categorized according to the protein function predicted by the KEGG database, and then the number of genes in each pathway was calculated. The KEGG pathways with significant enrichment of DEGs compared with the genomic background were defined using the hypergeometric test with the same conditions. This analysis predicted the cellular and metabolic functions associated with the observed changes in transcript levels.

### Quantitative real-time PCR analysis

To validate the RNA-seq results, samples from the same treatment and RNA extraction for RNA-seq were also used for cDNA synthesis. The RNA was reverse-transcribed into cDNA using the PrimeScript RT Reagent Kit with gDNA Eraser (Takara, Shiga, Japan). A total of 20 DEGs that differed in their expression pattern were verified by quantitative real-time PCR (qRT-PCR). The selected DEGs were mostly associated with sugar metabolism, alternative carbon metabolism, glutathione metabolism, and plant–pathogen interactions. Primers for the selected DEGs were designed using Primer Premier v5.0 (Premier Biosoft, San Francisco, CA, USA). The *P. olsonii* β*-actin* (POLN_004133) gene was used as the internal control gene for each amplification. All primers used are listed in Supplementary Table 1. Each qRT-PCR analysis was performed using a Bio-Rad CFX-96 Real-Time PCR System (Bio-Rad Laboratories, Hercules, CA, USA). Reactions were conducted with a final volume of 10 μl, containing 1.0 μl of diluted cDNA template, 0.5 μl of each forward and reverse primer (1 μM), 5 μl of SYBR^®^ Premix Ex Taq™ (Takara), and 3 μl of RNase-free water. The PCR protocol was as follows: initial denaturation at 96°C for 1 min, followed by 40 cycles at 96°C for 15 s, and primer extension at 62°C for 25 s. The relative expression level of each targeted gene was calculated using the 2^–△△^*^C^*^t^ method. Three replicates of each qRT-PCR analysis were performed for each gene.

### Observation of *P. olsonii* micromorphology after exposure to *B. velezensis* metabolites

Plates containing PDA medium were inoculated with 100 μl of WHG5 cell suspension and incubated at 28°C for 24 h. A small plug of the culture (diameter 5 mm) was inoculated onto plates containing PDA supplemented with either 10% *B. velezensis* metabolites or the equivalent volume of sterile distilled water (the control) and cultured at 28°C for 72 h. The micromorphology of *P. olsonii* conidia and mycelia was observed with a light microscope (DM500, Leica, Wetzlar, Germany).

### Assay for the quantitative determination of reduced glutathione and trehalose contents and generation of intracellular reactive oxygen species

Reduced glutathione was quantified in accordance with the spectrophotometric assay method of Tietze (1969). GSH reacts with sulfhydryl reagent 5,5′-dithio-bis (2-nitrobenzoic acid) (DTNB) and forms a complex with a characteristic absorption peak at 412 nm. Trehalose content was determined using the modified method of Vandercammen et al. (1990). The amount of reducing sugar produced by the catalysis of trehalose by phenol was determined using the 3,5-dinitrosalicylic acid method in which brownish-red amino compounds are formed with a characteristic absorption peak at 620 nm. The generation of intracellular reactive oxygen species (ROS) was assessed using the ROS-sensitive fluorescence indicator 2′,7′-dichlorodihydrofluorescein diacetate following the modified method of Goto et al. (1993).

### Statistical analysis

To ensure the validity of the experimental data and to reduce the variance, all tests were performed in triplicate. All statistical analyses were performed with GraphPad Prism v7.0 (GraphPad Software, San Diego, CA, USA). The significance of differences between the means of the treatments was analyzed with one-way ANOVA followed by paired-sample *t*-tests at the 5% significance level (*P* < 0.05).

## Results

### Sequencing and assembly of genomic data

**FIGURE 1:**
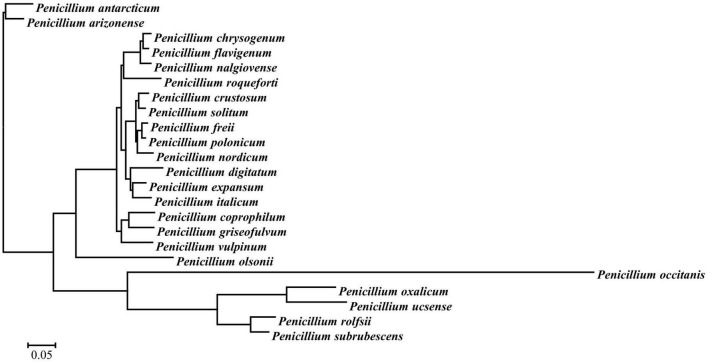
Phylogenetic tree for selected single-copy orthologous sequences in the genomes of *Penicillium olsonii* WGH5 and 23 other *Penicillium* species.

A total of 4,972 Mb of sequence data were generated for *P. olsonii* WHG5 (Table 1). The estimated genome size was 29.96 Mb, which was revised to 29.95 Mb, with an N50 value of 1,105,645 bp. Through comparison with publicly available databases, 10,251 protein-coding genes were predicted in the *P. olsonii* genome. The length of non-coding RNA was 24,188 bp in total. The genome assembly is summarized in Table 1. The assembled *P. olsonii* genome comprised a total length of 16,679,273 bp. The integrity of the genome assembly was assessed with BUSCO using the hypocreales_odb10 dataset, which includes 4,494 single-copy genes from 50 species. The results showed that 97.1% of the total number of single-copy genes were fully aligned in the WHG5 genome sequence (Table 1). In general, a high percentage implies high quality of genome assembly integrity. In addition, genome protein sequences for 23 species of *Penicillium* were clustered with the selected single-copy ortholog sequences in the WHG5 genome and they yielded 2,462 single copies. After a BLAST search of the ortholog library, the remaining 419 single-copy genes were used for a neighbor-joining analysis. The dendrogram (Figure 1) indicated that the WHG5 genome was most similar to the genomes of other *Penicillium* species.

**TABLE 1:** Genome assembly and annotation statistics of *Penicillium olsonii* WHG5.

Sample name	*Penicillium olsonii*	
**Assembly statistics**		
Scaffold total length (bp)	29,294,745	
Scaffold N50 length (bp)	1,105,645	
Scaffold N90 length (bp)	517,735	
GC content (%)	49.65	
**Gene statistics**		
Gene assembly (bp)	16,679,273	
Number of coding sequence genes	10,251	
Number of exons	31,134	
Total exons length (bp)	15,298,532	
Average length of coding sequence genes (bp)	1,627	
Average exons length (bp)	491.3	
Average introns length (bp)	66.1	
**BUSCO statistics**	**Numbers**	**Percent (%)**
Complete BUSCOs	3,928	97.1
Complete and single-copy BUSCOs	3,915	96.8
Complete and duplicated BUSCOs	13	0.3
Fragmented BUSCOss Missing BUSCOs	37	0.9
Missing BUSCOs	81	2.0
Total BUSCO groups searched	4,046	
**Genome functional annotation**	**Numbers**	**Percent (%)**
NR	10,003	97.58
GO	7,242	70.65
KEGG	4,003	39.05
KOG	9,544	93.10
SwissProt	7,589	74.03
CAZy	583	5.70

**FIGURE 2:**
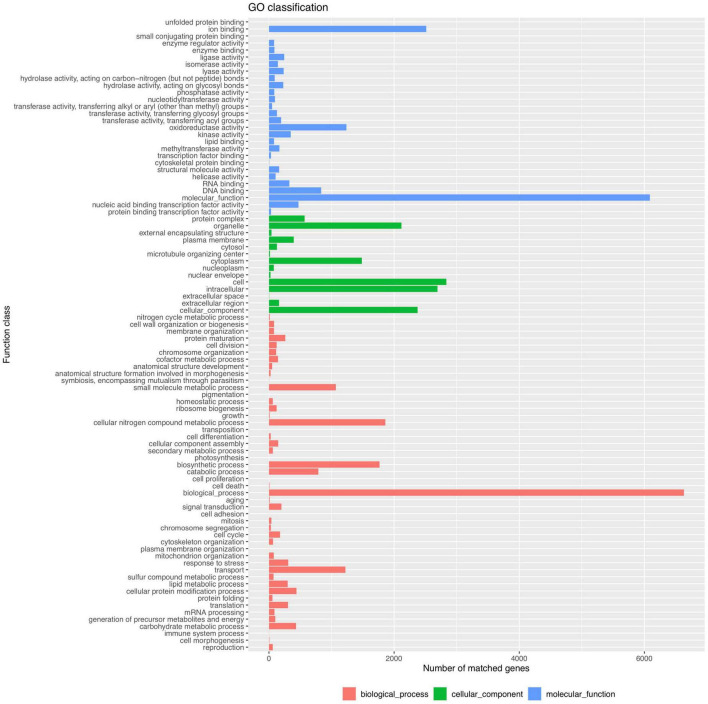
Gene ontology (GO) functional classification of the *Penicillium olsonii* WGH5 genome.

**FIGURE 3:**
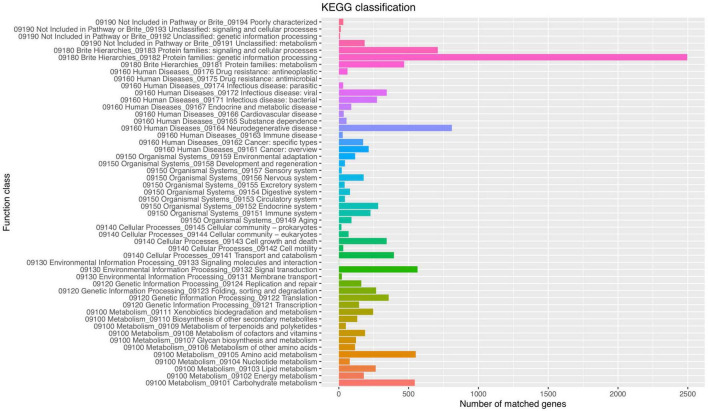
Kyoto encyclopedia of genes and genomes (KEGG) pathway functional classification of the *Penicillium olsonii* WGH5 genome.

Functional analysis of the *P. olsonii* WHG5 genome was performed with the consideration of GO classification (Figure 2), KEGG pathways (Figure 3), and the eggNOG classification (Supplementary Figure 2). The GO-based classification revealed a total of 7,242 annotated genes in the WHG5 genome. Among genes classified in the biological process and molecular function categories, cell (2,893, 39.95%), ion binding (2,514, 34.71%), and cellular nitrogen compound metabolic process (1,860, 25.68%) were the most abundant. Among genes classified in the cellular component category, the greatest number of genes were assigned to the intracellular (2,694, 37.20%), organelle (2,118, 29.25%), and cytoplasm (1,485, 20.51%) terms. The KEGG database indicated that the most abundant genes were associated with genetic information (2,496, 62.35%), neurodegenerative disease (810, 20.23%), and signaling and cellular process (709, 17.71%) pathways. These findings suggested the presence of genes involved in a variety of protein metabolism, energy metabolism, and signal transduction processes in *P. olsonii*. Similarly, the most gene-rich KOG functional terms were carbohydrate transport and metabolism (817, 8.56%); amino acid transport and metabolism (530, 5.55%); and post-translational modification, protein turnover, and chaperones (490, 5.13%). In addition, carbohydrate-active enzymes (CAZymes) were important in the *P. olsonii* WHG5 genome (Supplementary Figure 3).

### Evaluation of the transcriptome data

In our preliminary study, metabolites produced by *B. velezensis* SB023 significantly inhibited the growth of *P. olsonii* after culturing for 72 h. In the present study, *P. olsonii* cultured in the presence or absence of SB023 metabolites was subjected to RNA-seq to screen for potential candidate genes involved in the interaction between *B. velezensis* and *P. olsonii*. After the removal of low-quality reads, the number of clean reads ranged from 6.72 to 6.86 Gb. The bases with Q ≥ 30 comprised more than 95.50% of the total number and those with Q ≥ 20 comprised more than 98.43% (Supplementary Table 2). More than 91.79% of the clean RNA-seq data after quality control were mapped to the *P. olsonii* WHG5 reference genome, indicating that the quality of the transcriptome sequence data was reliable (Supplementary Table 2).

**FIGURE 4:**
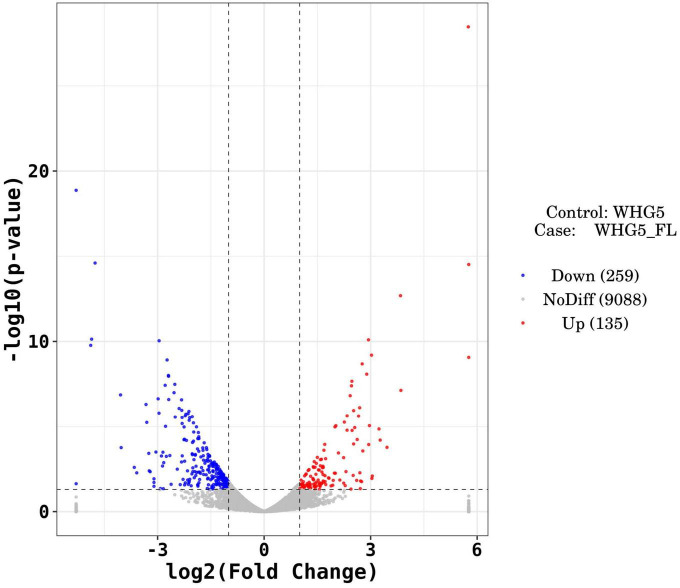
Differentially expressed genes (fold change > 1) between *Penicillium olsonii* WHG5 (control; WHG5 cultured without *Bacillus velezensis* metabolites) and WHG5_FL (treatment; WHG5 cultured with *B. velezensis* metabolites). Down-regulated genes are in blue, up-regulated genes are in red, and non-differentially expressed genes are in gray.

**FIGURE 5:**
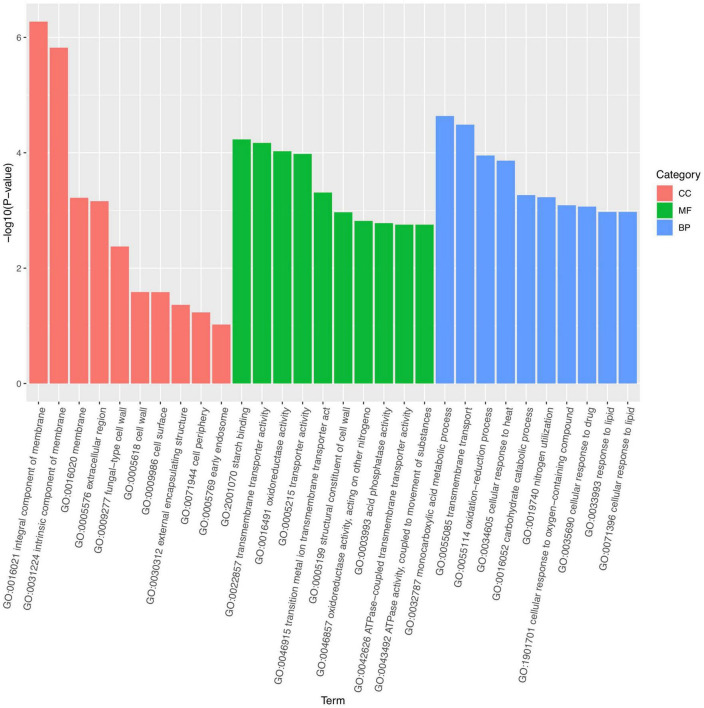
Gene ontology (GO) functional annotation and classification of expressed genes (fold change > 1) between *Penicillium olsonii* WHG5 (control; WHG5 cultured without *Bacillus velezensis* metabolites) and WHG5_FL (treatment; WHG5 cultured with *B. velezensis* metabolites).

**FIGURE 6:**
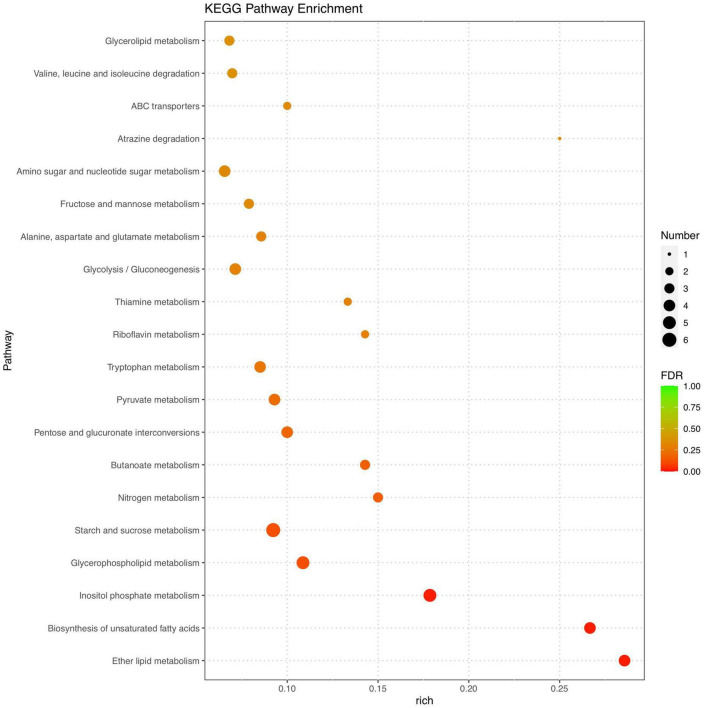
Kyoto encyclopedia of genes and genomes (KEGG) pathway functional annotation of differentially expressed genes (fold change > 1) between *Penicillium olsonii* WHG5 (control; WHG5 cultured without *Bacillus velezensis* metabolites) and WHG5_FL (treatment; WHG5 cultured with *B. velezensis* metabolites).

Marked differences in the transcriptome profiles were observed between *P. olsonii* WHG5 cultured in the presence or absence of *B. velezensis* metabolites. A total of 394 core DEGs were obtained. Compared with the control group (WHG5 cultured without *B. velezensis* metabolites), 135 and 259 DEGs were up-regulated and down-regulated, respectively, in WHG5 cultured with *B. velezensis* metabolites (Figure 4). To identify the biological processes, molecular functions, and cellular components of WHG5 growth influenced by *B. velezensis* metabolites, a GO term analysis was conducted on the selected DEGs (Figure 5). In the cellular process category, the most abundant GO terms were an integral component of the membrane, an intrinsic component of the membrane, and the membrane. In the metabolic process category, the predominant GO terms comprised the starch binding, transmembrane transporter activity, and oxidoreductase activity. In the biological process category, the predominant GO terms comprised monocarboxylic acid transport, transmembrane transport, and oxidation–reduction process. The KEGG pathway analysis revealed that the DEGs mostly participated in lipid metabolism, biosynthesis of unsaturated fatty acids, inositol phosphate metabolism, glycerophospholipid metabolism, starch and sucrose metabolism, and nitrogen metabolism (Figure 6). The GO and KEGG analyses implied that *B. velezensis* metabolites influenced specific metabolic processes in *P. olsonii*. Notably, most of the genes in *P. olsonii* involved in starch and sucrose metabolism and oxidative phosphorylation were differentially expressed after exposure to *B. velezensis* metabolites.

### Analyses of differentially expressed genes involved in sugar metabolism

**FIGURE 7:**
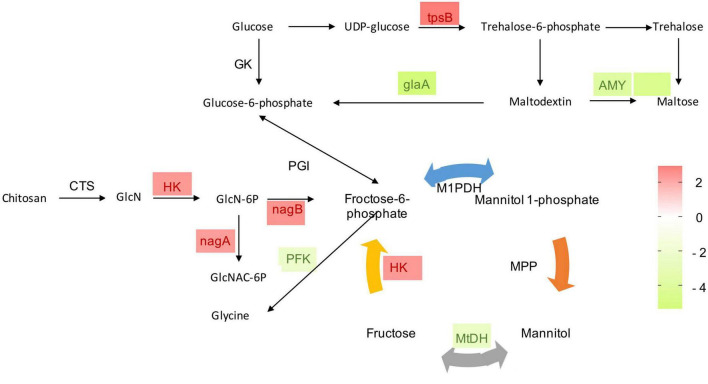
Heat maps of differentially expressed genes (DEGs) that may be involved in sugar metabolism. GK, glucokinase; PGI, phosphoglucose isomerase; MtDH, mannitol-2-dehydrogenase; HK, hexokinase; M1PDH, mannitol-1-phosphate dehydrogenase; MPP, mannitol-1-phosphate phosphatase; tpsB, trehalose 6-phosphate synthase; AMY, alpha-amylase A; gLaA, glucoamylase; CTS, chitosanase; GlcN, glucosamine; GlcN-6P, glucosamine 6-phosphate; GlcNAc-6P, 6-phosphate-*N*-acetylglucosamine; nagA, 6-phosphate-*N*-acetylglucosamine deacetylation; nagB, 6-phosphate glucosamine deaminase; PFK, phosphofructokinase. The expression image was generated using GraphPad Prism software: red, increase; green, decrease.

Carbon utilization and metabolism are fundamental for cellular growth in every living organism. Therefore, glycolysis is vital for normal growth and metabolism. In the present study, mannitol metabolism starting with fructose-6-phosphate, and trehalose synthesis starting with glucose-6-phosphate of *P. olsonii* were affected by *B. velezensis* metabolites (Figure 7). Genes encoding key enzymes in these sugar metabolic pathways were differentially expressed in response to exposure to *B. velezensis* metabolites. Among these DEGs, a gene encoding mannitol-1-phosphate dehydrogenase (MtDH; POLN_001215), a crucial enzyme in the interconversion of mannitol and fructose, was down-regulated in the treatment group. Genes encoding alpha-amylase A (AMY; POLN_001517 and POLN_001645) and glucoamylase (glaA; POLN_001644) showed the same pattern. AMY1 is essential for the synthesis of a virulence factor and hence is associated with virulence. In addition, DEGs encoding other crucial enzymes were up-regulated in the treatment group, including 6-phosphate-*N*-acetylglucosamine deacetylation (nagA; POLN_005457), 6-phosphate glucosamine deaminase (nagB; POLN_005454), and hexokinase (HK; POLN_002794), which are key enzymes involved in the breakdown of fructose and other sugars into fructose-6-phosphate for glycolysis. It was notable that the up-regulated enzymes also included trehalose 6-phosphate synthase (tpsB; POLN_002453), which is a critical enzyme in trehalose synthesis. In addition, the trehalose content was significantly increased (*P* < 0.05) in the treatment group (Table 2).

**TABLE 2:** Contents of glutathione, trehalose, and reactive oxygen species in *Penicillium olsonii* WHG5.

	Control group	Treatment group
Glutathione (μmol/g fresh weight)	0.377 ± 0.017	0.875 ± 0.020
Trehalose (mg/g fresh weight)	5.750 ± 0.220	6.426 ± 0.218
Reaction oxygen species generation rate (u/s/g fresh weight)	3055.546 ± 89.418	1131.889 ± 63.653

### Analyses of differentially expressed genes involved in alternative carbon metabolism

**FIGURE 8:**
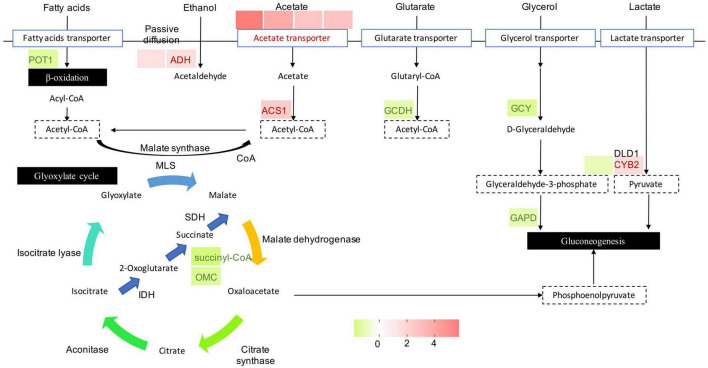
Heat maps of differentially expressed genes (DEGs) that may be involved in alternative carbon metabolism. POT, 3-ketoacyl-CoA thiolase; ADH, alcohol dehydrogenase; ACS, acetyl-CoA synthetase; GCDH, glutaryl-CoA dehydrogenase; GCY, glycerol 2-dehydrogenase; GAPD, glyceraldehyde-3-phosphate dehydrogenase; DLD, dihydrolipoamide dehydrogenase; CYB2, cytochrome b2; OMC, 2-oxoglutarate-malate carrier; SDH, succinate dehydrogenase; MLS, malate synthase; IDH, isocitrate dehydrogenase. The expression image was generated using GraphPad Prism software: red, increase; green, decrease.

As an important metabolic adaptation strategy, alternative carbon metabolism is utilized by a fungus to compensate for the deficiency of the preferred carbon source, glucose. In such a harsh microenvironment, the fungus converts other carbon sources to the central metabolite acetyl-CoA to fuel the glyoxylate cycle and gluconeogenesis for glucose and energy production. After exposure of *P. olsonii* to *B. velezensis* metabolites for 5 days, 15 DEGs encoded critical enzymes in the glyoxylate cycle and alternative carbon metabolism (Figure 8). Different sources of acetyl-CoA responded differently to *B. velezensis* metabolites. Among these DEGs, genes encoding 3-ketoacyl-CoA thiolase (POT1; POLN_005154) involved in β-oxidation of fatty acids, glutaryl-CoA dehydrogenase (GCDH; POLN_006747) involved in glutamate metabolism, glycerol 2-dehydrogenase (GCY1; POLN_008918) and glyceraldehyde-3-phosphate dehydrogenase (GAPD; POLN_006304) involved in glycerol metabolism, and cytochrome b2 (CYB; POLN_005941) involved in lactate metabolism were down-regulated in the treatment group. Conversely, key genes in other sources of acetyl-CoA were up-regulated in the treatment group, including DEGs encoding alcohol dehydrogenase (ADH; POLN_000033 and POLN_000551) involved in ethanol metabolism, and acetyl-CoA synthetase (ACS; POLN_000098) and related acetyltransferases (POLN_000095, POLN_006411, POLN_002030, and POLN_004443) involved in acetate metabolism. These results implied that *B. velezensis* metabolites influenced the β-oxidation of fatty acids, glutamate metabolism, glycerol metabolism, and lactate metabolism, and increased the metabolism of ethanol and acetate in *P. olsonii*. In the glyoxylate cycle, isocitrate is converted to malate through the glyoxylate process and utilizes acetyl-CoA. It is worth mentioning that two DEGs encoding key proteins in this process, succinyl-CoA (POLN_006914) and 2-oxoglutarate-malate carrier (OMC; POLN_009692), were down-regulated in the treatment group, which indicated that isocitrate conversion to malate through the succinate process was decreased by *B. velezensis* metabolites.

### Analyses of differentially expressed genes involved in the maintenance of redox homeostasis

**FIGURE 9:**
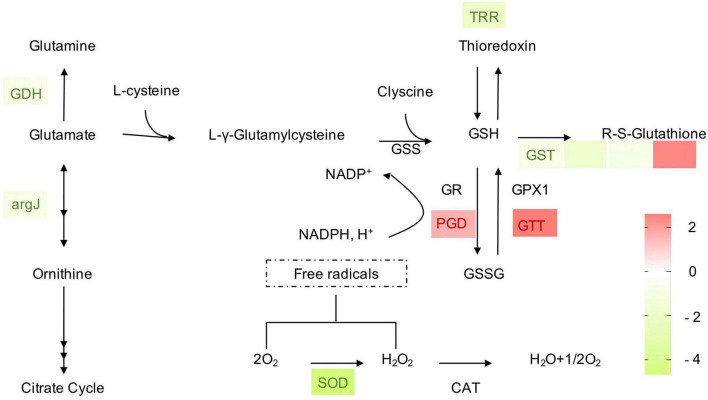
Heat maps of differentially expressed genes (DEGs) that may be involved in redox homeostasis. GDH, glutamate dehydrogenase; argJ, amino-acid acetyltransferase; GSS, glutathione synthase; GSH, glutathione; GR, glutathione reductase; PGD, 6-phosphogluconate dehydrogenase; GPX, glutathione peroxidase; GTT, glutathione transporter; GSSG, glutathione disulfide; GST, glutathione *S*-transferase; TRR, thioredoxin reductase; SOD, superoxide dismutase; CAT, catalase. The expression image was generated using GraphPad Prism software: red, increase; green, decrease.

For fungal adaptation to an unfavorable environment, enzymatic [catalases, superoxide dismutases (SODs), and peroxidases] and non-enzymatic [glutathione (GSH)] mechanisms maintain the redox homeostasis within the cells. In these processes, the oxidation status is maintained and rapidly restored by the action of two redox-balancing systems: the GSH and thioredoxin pathways. In the present study, DEGs were enriched in the GSH pathway, indicating that *B. velezensis* metabolites affected GSH metabolism in *P. olsonii* (Figure 9). Four DEGs encoding glutathione *S*-transferases (GST; POLN_004024, POLN_001793, POLN_004336, and POLN_007150) were identified in the treatment group, of which one (POLN_004024) was up-regulated. As the main source of GSH, glutamate synthesis was affected in the treatment group by the down-regulation of the genes encoding glutamate dehydrogenase (GDH; POLN_000199) and amino-acid acetyltransferase (argJ; POLN_004328). GDH converts glutamate into glutamine, whereas argJ converts glutamate into ornithine and participates in the citrate cycle. Two DEGs encoding enzymes that participate in the interconversion of GSH and GSSH, namely 6-phosphogluconate dehydrogenase (PDG; POLN_008064) and glutathione transporter (GTT; POLN_002687), were up-regulated in the treatment group, indicating that interconversion between GSH and GSSH in *P. olsonii* was activated in response to exposure to *B. velezensis* metabolites. A gene encoding thioredoxin reductase (TRR; POLN_000490) was down-regulated in the treatment group, indicating that thioredoxin metabolism was decreased. In addition, the glutathione content was increased and intracellular ROS generation was reduced in the treatment group (*P* < 0.05) (Table 2). However, a gene encoding SOD (POLN_000741), a vital enzyme that maintains the redox homeostasis within the cell, was down-regulated in the treatment group, which indicated that *B. velezensis* metabolites depressed the growth of *P. olsonii*.

### Analyses of differentially expressed genes involved in plant–pathogen interactions

**FIGURE 10:**
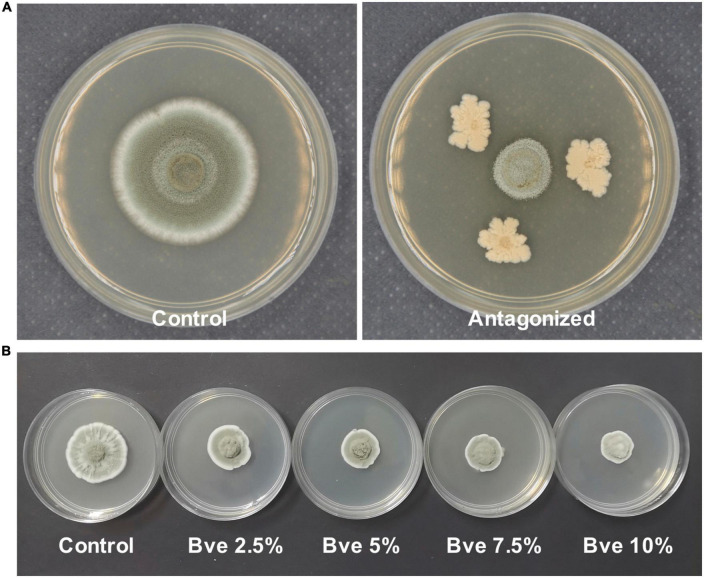
Morphology of *Penicillium olsonii* on potato dextrose broth (PDA) after exposure to *Bacillus velezensis* (5 days after inoculation). Antagonized by *B. velezensis* on PDA **(A)**, colony of *P. olsonii* after exposure to different concentrations of *B. velezensis* metabolites **(B)**.

**FIGURE 11:**
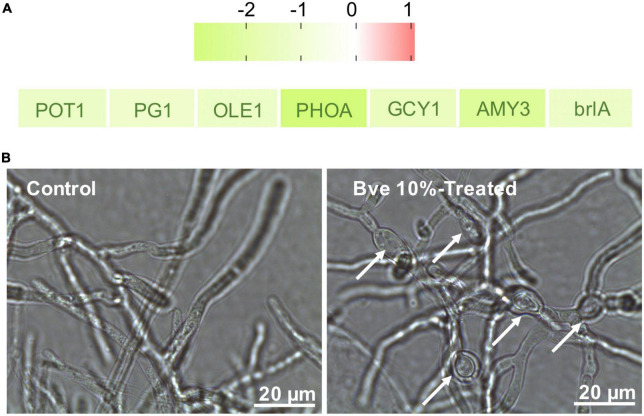
Identification of differentially expressed genes (fold change > 1) involved in plant–pathogen interactions by RNA sequencing **(A)**. Microscopic morphology of *Penicillium olsonii* after exposure to *Bacillus velezensis* (5 days after inoculation) metabolites. White arrows indicate mycelial swelling caused by 10% *B. velezensis* metabolites **(B)**.

Although we only observed the growth of *P. olsonii* on PDA plates supplemented with *B. velezensis* metabolites (Figure 10), the transcript data revealed that several DEGs were involved in plant–pathogen interactions (Figure 11A). In addition to *POT1* mentioned with regard to alternative carbon metabolism, DEGs encoding polygalacturonase 1 (PG1; POLN_000891), stearoyl-coenzyme A desaturase 1 (OLE1; POLN_002255), and phosphate-repressible acid phosphatase (PHOA; POLN_005738) were down-regulated in the treatment group. PG1 is a key virulence protein of post-harvest pathogens, OLE1 is associated with fungal virulence, and PHOA is one of the main toxins of *Penicillium* species. Thus, *B. velezensis* metabolites may affect the pathogenicity of *P. olsonii*. In addition, given that spore formation requires the participation of mannitol, genes associated with sporulation were down-regulated in the treatment group, including *GCY1* and *AMY3* mentioned in relation to carbon metabolism, *PFK*, and *brlA* (regulatory protein, POLN_005970), a gene associated with conidia formation. These results indicated that *B. velezensis* metabolites may alter the normal sporulation of *P. olsonii*. To verify these results, the development of *P. olsonii* spores and mycelia was observed microscopically. The conidial spores were deformed and the swelling of the mycelia of *P. olsonii* was observed after exposure to *B. velezensis* metabolites (Figure 11B).

### Validation of RNA-seq results by qRT-PCR analysis

**FIGURE 12:**
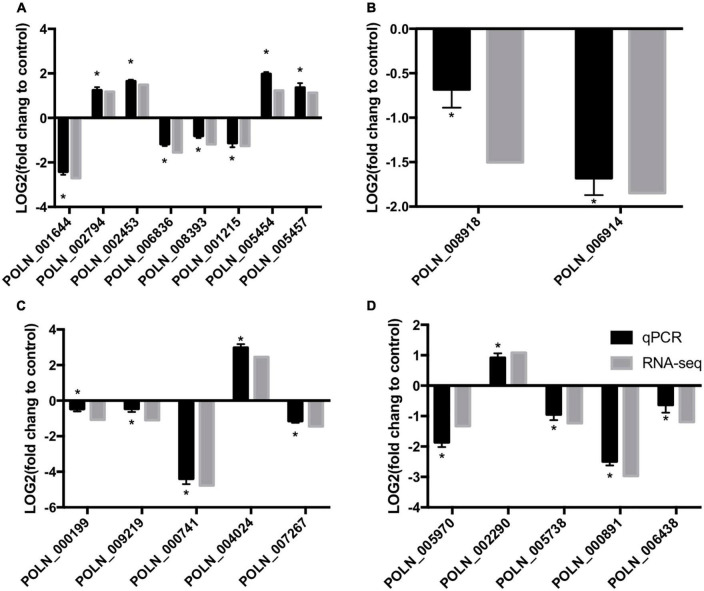
qRT-PCR verification of RNA-sequencing results with genes involved in sugar metabolism **(A)**, alternative carbon metabolism **(B)**, glutathione metabolism **(C)**, and plant–pathogen interactions **(D)**. Log_2_(fold change) > 0 represents up-regulation of gene expression, Log_2_(fold change) < 0 represents down-regulation of gene expression. An asterisk above an error bar indicates a significant difference between the treatment group and the control group (*P* < 0.05).

To verify the changes in gene expression detected from the RNA-seq analysis, 20 DEGs that differed in expression pattern were quantified by qRT-PCR. The expression levels of almost all up- and down-regulated genes corresponded with the RNA-seq results (Figure 12). However, one DEG (POLN_000199) was down-regulated in the treatment group according to the RNA-seq analysis, but it showed a slight increase in expression in the treatment group according to the qRT-PCR analysis. These results verified the overall reliability of the RNA-seq data.

## Discussion

The post-harvest storage life of fruit may be reduced as a result of infection by a variety of fungal pathogens. As a typical fresh fruit, the post-harvest shelf life of grape berries is greatly affected by pathogens. Considering the importance of food safety, eco-friendly and effective measures are urgently needed to control post-harvest diseases of grapes. *B. velezensis* and its metabolites show potential as an eco-compatible alternative to the application of chemical fungicides (Rabbee et al., 2019; Fazle Rabbee and Baek, 2020). Our preliminary study indicated that metabolites of *B. velezensis* may control *P. olsonii* infection of grape berries by direct inhibition of *P. olsonii* growth (Figure 10). Research is increasingly focused on the indirect induction of disease resistance in grapes in response to pathogen infection, but how metabolites directly inhibit the growth of post-harvest pathogens is uncertain (Fan et al., 2018; Rabbee et al., 2019). Hence, we conducted an analysis of transcriptomic changes in *P. olsonii* isolate WHG5 after exposure to *B. velezensis* metabolites. The results confirmed that *B. velezensis* metabolites offer a promising strategy for the effective management of post-harvest *Penicillium* spp. to suppress post-harvest spoilage of grape berries.

The present results revealed that exposure to *B. velezensis* metabolites caused up-regulated expression of genes encoding the crucial enzymes (HK, nagB, and nagA) that generate fructose-6-phosphate, a central metabolite in sugar metabolism. This finding is consistent with previous research on fungi under stress (Ram, 2004; Arora et al., 2020). By affecting sugar metabolism in *P. olsonii*, treatment with *B. velezensis* metabolites might lead to energy deficiency in the pathogen. The decreased energy status would further accelerate the metabolism of energy-associated substances, and the disturbance of energy metabolism might ultimately accelerate cell death. Mannitol has generally been considered to function as a carbohydrate reserve, but more recently, it has been hypothesized to play a major role in fungal pathogenicity (Patel and Williamson, 2016). Double-knockout mutants (*Dmtdh Dm1pdh*) of *A. alternata* and *A. brassicicola*, which are deficient in mannitol synthesis, show greatly reduced virulence in tobacco and cabbage, respectively (Calmes et al., 2013). In the present research, the down-regulation of *MtDH* expression and up-regulation of *HK* expression may cause a decrease in mannitol content in *P. olsonii*. Moreover, an increase in *tasB* expression level and the down-regulation of *AMY* and *glaA* expression resulted in significantly higher contents of trehalose in the treatment group. Given that trehalose is a reserve carbohydrate (Li and Tian, 2007; Apaliya et al., 2018), the present results suggested that acceleration of sugar metabolism and increased activity of alternative carbon metabolism in *P. olsonii* were induced by *B. velezensis* metabolites. These conclusions were supported by the up-regulated expression of two genes (*ADH* and *ACS*) involved in alternative carbon metabolism, including ethanol metabolism and acetate metabolism (Fujino et al., 2001; Cobos et al., 2010). However, the activity of other alternative carbon metabolism pathways, such as β-oxidation of fatty acids, glutamate metabolism, glycerol metabolism, and lactate metabolism, was decreased by exposure to *B. velezensis* metabolites, as indicated by the down-regulated expression of genes encoding key enzymes involved in these pathways (POT1, GCDH, GCY, and GAPD) (Delgado et al., 2001; Ortiz et al., 2017; Weber et al., 2020; Zhang et al., 2021a). Hence, the present results showed that *B. velezensis* metabolites affected normal carbon metabolism in *P. olsonii* and may induce the pathogen to utilize alternative carbon metabolism through ethanol metabolism and acetate metabolism, which partly explained the antifungal activity of *B. velezensis* metabolites.

Glutathione metabolism in a fungus might play a pivotal role in amino acid transport, regulation of enzyme activity, and the maintenance of the tissue redox homeostasis, all of which contribute to the protection of the fungus against plant-derived toxic metabolites and the accumulation of ROS during infection at the host–pathogen interface (Gullner et al., 2018; Khullar and Reddy, 2018). The reduction of ROS is considered to follow the destruction of mitochondria, and ROS signals are required for apical dominance and development of the fungus (Hayashi et al., 2014; Sun et al., 2016). The decreased production of intracellular ROS in the treatment group indicated the destruction of *P. olsonii* caused by *B. velezensis* metabolites. In addition, multiple genes associated with the glutathione cycle (GST, GTT, PGD, and GDH) were differentially expressed in *P. olsonii* in response to exposure to *B. velezensis* metabolites. These changes were indicative of the damage to *P. olsonii* caused by *B. velezensis* metabolites and the compensatory responses of *P. olsonii* to this adverse stress. Treatment with *B. velezensis* metabolites decreased the expression levels of genes encoding GST, TRR, GDH, argJ, and SOD, and increased the content of reduced glutathione, which restricted the reduction and utilization of glutathione and the scavenging of free radicals, and thus may have disrupted the redox homeostasis in *P. olsonii*. *P. olsonii* responded to the stress caused by *B. velezensis* metabolites by up-regulating the expression of genes encoding PDG and fructose-bisphosphate aldolase, which are important enzymes in the pentose phosphate pathway (Rojas et al., 2014). This pathway provides NADPH without consuming ATP for the production of cellular fatty acids and reduced glutathione, thereby promoting the survival of the organism under stress (Rojas et al., 2014; Campbell et al., 2016). Notably, GST is also associated with pathogen virulence (Calmes et al., 2015; Gullner et al., 2018). Thus, the down-regulation of genes encoding GST in *P. olsonii* in the treatment group further demonstrated the potential for *B. velezensis* metabolites to suppress post-harvest spoilage in grapes.

Previous studies on biocontrol mechanisms have indicated that *B. velezensis* may suppress the growth and spore formation of pathogens by secreting certain secondary metabolites or releasing a number of volatile organic compounds (Calvo et al., 2020; Li et al., 2020). Scanning electron microscopic observation of *Penicillium roqueforti* conidiospores exposed to purified iturin A revealed the destruction and morphological changes in the spores, and the assessment of spore inactivation by fluorescence techniques indicated that iturin A may permeabilize the spores and inhibit their germination (Calvo et al., 2020). In the current study, in addition to the observation that *B. velezensis* metabolites disrupted the mycelial growth of *P. olsonii*, the expression of genes associated with mycelial and spore growth was down-regulated (Figure 11). Among these genes, the down-regulation of *brlA* and *abaA* in *P. olsonii* in response to exposure to *B. velezensis* metabolites was noteworthy. Deletion of *PdbrlA* in *Penicillium digitatum* completely inhibited conidiophore development, whereas the deletion of *PdabaA* led to the formation of aberrant and non-functional phialides (Wang et al., 2015).

Several genes involved in plant–pathogen interactions were down-regulated in *P. olsonii* after exposure to *B. velezensis* metabolites. This response would enhance the reliability of *B. velezensis* metabolites as a potential biocontrol agent to suppress post-harvest spoilage in grapes. Given that the plant cell wall is a crucial physical barrier in plant–fungal pathogen interactions, the degradation of major components of the cell wall is vitally important for fungal infection (Underwood, 2012). In particular, pectinolytic enzymes are more important in the case of soft-rotting post-harvest pathogens than in other plant–fungus interactions (Reignault et al., 2007). Down-regulation of *PG1* in *P. olsonii* indicated the potential ability of *B. velezensis* metabolites to reduce the pathogenicity of *P. olsonii*. Moreover, the gene encoding the mycotoxin phomopsin A (*PHOA*) was down-regulated in *P. olsonii* after exposure to *B. velezensis* metabolites. PHOA is the primary toxin among the group of secondary metabolites formed by the fungus (Kunz et al., 2022). Therefore, the down-regulation of *PHOA* in *P. olsonii* caused by *B. velezensis* metabolites is an additional indication of the potential of *B. velezensis* metabolites as an eco-compatible alternative to harmful chemical fungicides.

## Conclusion

*Bacillus velezensis* metabolites inhibit mycelial growth and cause the deformation of the conidia of the grape post-harvest pathogen *P. olsonii* WHG5. Genomic and transcriptomic analyses indicate that *B. velezensis* metabolites affect the expression of genes that influence the mycelial growth of *P. olsonii*. The inhibition of mycelial growth might be due to an initial impact on normal sugar metabolism and subsequent disturbance of alternative carbon metabolism and the stimulation of glutathione metabolism to maintain redox homeostasis. Although *P. olsonii* was grown on a PDA medium in this study, genes involved in plant–pathogen interactions were down-regulated by *B. velezensis* metabolites, which further verified the effect on the pathogenicity of *P. olsonii*. The results provide an overview of the main responses of *P. olsonii* to *B. velezensis* metabolites and highlight potential target genes for biocontrol agents to suppress post-harvest spoilage in grapes.

## Data availability statement

The data presented in this study are deposited in the NCBI SRA repository, accession numbers SRX17100770 and SRX17100771.

## Author contributions

TZ performed the experiments and wrote the original draft of the manuscript with the assistance of GW and SJ. BS and ZC carried out data curation and participated in the revision of the manuscript. SJ designed the research and provided financial support. All authors have read and approved the submitted version of the article.

## Footnotes

1
http://www.ebi.ac.uk/about/vertebrate-genomics/software

2
http://www.genome.jp/kegg/
