# Supplementary material for: Transcriptome profiling reveals the underlying mechanism of grape post-harvest pathogen Penicillium olsonii against the metabolites of Bacillus velezensis
Source: Front Microbiol. 2023 Jan 18;13:1019800. doi: 10.3389/fmicb.2022.1019800 (PMC9889648; doi:10.3389/fmicb.2022.1019800)
Supplement: Supplementary file 1 [file Table_1.docx]

Supplementary Material

Supplementary Table S1| Primers of slected DEGs for qRT-PCR

| Gene ID | Forward primer | Reverse primer |
| --- | --- | --- |
| POLN_008918 | CCAACATCGGTGTCTCCAAC | AACCTGCTGAGGAGTCTTGC |
| POLN_005970 | ATGGACCGCCAAGAGCAAAT | GTGGTAGTAGTTGCGAGCC |
| POLN_002290 | GCTGCTACCCACTCCATTC | GTTGAGGGAAAGAACCACGC |
| POLN_005738 | CTTCGGTTACAAGGAGTGGA | TCCAGAGAGTGGGCGTAGAAG |
| POLN_006914 | GCCGTAACGCTAAGATGACC | TCAATTCCCTTGGGGGTGGT |
| POLN_000891 | CTGGTTCTGTTTGACTCCGT | AGGAACTTGGCAGACCACTCG |
| POLN_001644 | CAGGCATACATCCAAACCGT | AGCCACTGTCCGAATGAGAT |
| POLN_002794 | ACGGAAGTGGATTGGTGGTT | GCTGACCCGTCGTTCACAAT |
| POLN_002453 | ATCCAGCCATCCAGTCAAG | CGACCTTCTCACTCCATTCT |
| POLN_000199 | CAGCCCTTGTATGGTATGTT | GGTGTTTGCCAGGTTGTAGT |
| POLN_009219 | CCTGGCTACGCTGATTACAT | GGGATGGTATCGCCGTATGT |
| POLN_006836 | TGCCAAGAACATCATCATCC | CAATGCGAGCAGTGGTAACG |
| POLN_008393 | TGTCAACGCAACCGCCAAGT | ATGCTTTGCGAACTCCTTGG |
| POLN_001215 | TGCCTCGTGTCAGTCTCAAT | CGAGGATGTATCGGAACCAG |
| POLN_006438 | GGTCCGCTCTACAAGTCCAT | CCTGGAACAACCGCAACAC |
| POLN_000741 | TGTTCCCATCTTTGGTGTTG | CAACTCCAGCAGTGTATCGC |
| POLN_004024 | GTCACACTTCCCGACATCAC | GAACGAGTCGCCAATCTTAT |
| POLN_007267 | ACACCGTGGACATTGAACAG | CCACACTCGGAGCAGAAGAT |
| POLN_005454 | GTTCTTGGTCTGCCTACTGG | GTTGAGGATGTTGATGTTCTG |
| POLN_005457 | TGAACAAAGGGCTGGCTCG | GGACTTGTGGATACCGTTGC |
| POLN_004133 | ATGAGGCACAGTCCAAGCGT | CTTCTCACGGTTGGACTTGG |

Supplementary Table S2| Transcriptome statistics of *Penicillium olsonii* WHG5.

| Sample name | WGH5 | WGH5_FL |
| --- | --- | --- |
| **Evaluation statistics** |  | |
| Number of clean reads | 44,798,590 | 45,748,350 |
| Clean reads length (bp) | 6,719,788,500 | 6,862,252,500 |
| Clean reads (%) | 91.79 | 92.13 |
| Numbers of clean reads total mapped to WGH5 genome | 43,790,326 | 44,848,478 |
| Numbers of clean reads multiple mapped to WGH5 genome | 208,782 | 102,688 |
| Numbers of clean reads uniquely mapped to WGH5 genome | 43,581,544 | 44,745,790 |

Supplementary Figure S1| KOG function classification of P. olsonii WGH5 genome

Supplementary Figure S2| carbohydrate-active enzymes (CAZymes) in P. olsonii WGH5 genome
