# Supplementary figures and images for: Transcriptome profiling reveals the underlying mechanism of grape post-harvest pathogen Penicillium olsonii against the metabolites of Bacillus velezensis
Source: Front Microbiol. 2023 Jan 18;13:1019800. doi: 10.3389/fmicb.2022.1019800 (PMC9889648; doi:10.3389/fmicb.2022.1019800)

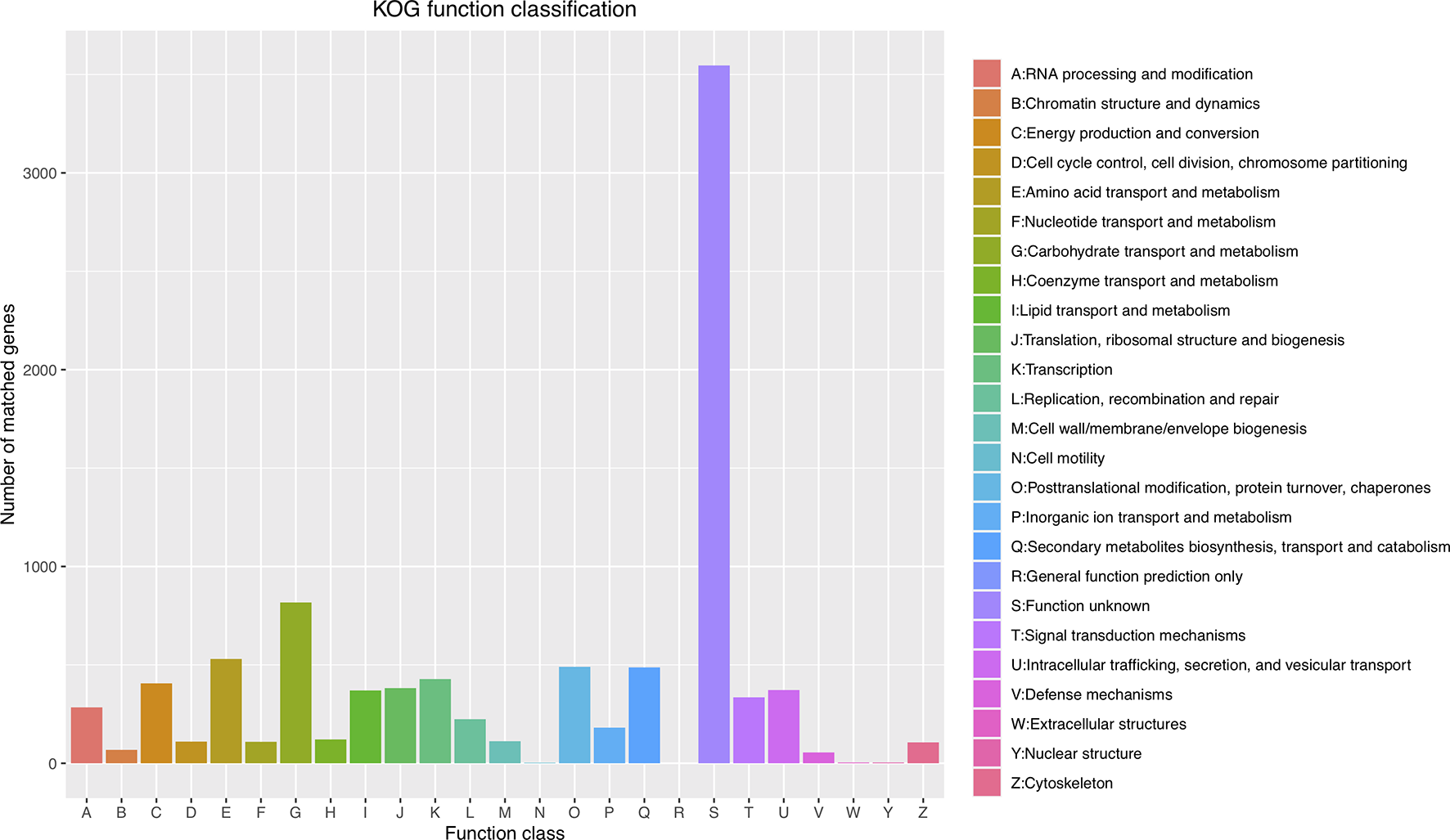

Supplement: Supplementary file 2 [file Image_1.TIFF]

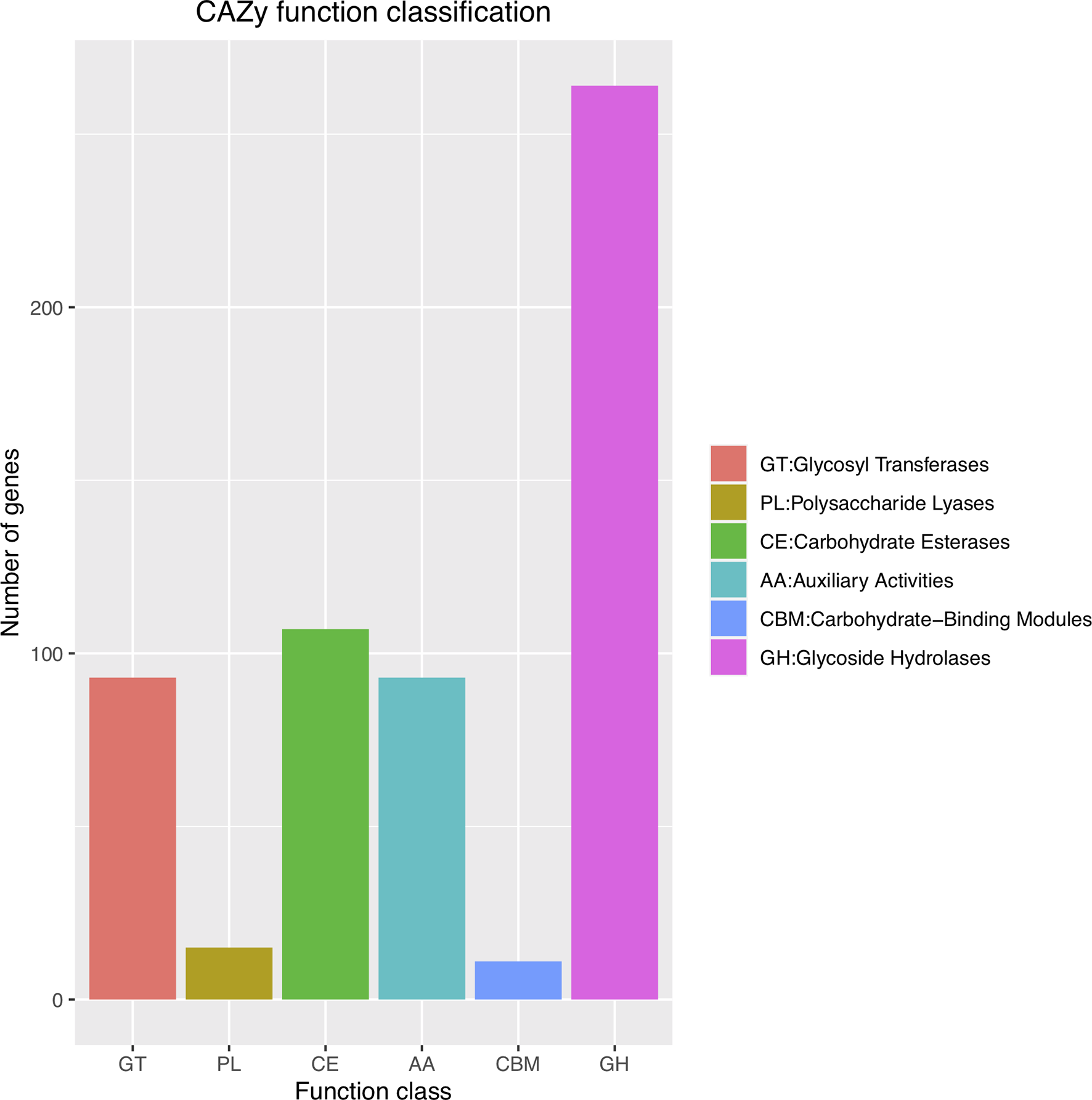

Supplement: Supplementary file 3 [file Image_2.TIFF]
